# Dosage Form Data Used for Estimating Pediatric Antibiotic Use

**DOI:** 10.3797/scipharm.1511-05

**Published:** 2015-07-01

**Authors:** Maria Matuz, Ria Benko, Monique Elseviers, Edit Hajdu, Peter Doro, Reka Viola, Gyongyver Soos

**Affiliations:** 1 Department of Clinical Pharmacy, Faculty of Pharmacy, University of Szeged, Szikra utca 8., H-6725 Szeged, Hungary; 2 Centre for Research and Innovation in Care (CRIC), Faculty of Medicine and Health Sciences, University of Antwerp, CDE R3.29, Universiteitsplein 1, B-2610 Wilrijk, Belgium; 3 First Internal Medicine, Infectiology Unit, Faculty of Medicine, University of Szeged, Kálvária sgt 57, H-6724 Szeged, Hungary

**Keywords:** Pediatric antibiotic use, New estimation method, Pharmacoepidemiology, Liquid oral formulations, Drug utilization research

## Abstract

We aimed to report a simple estimation method to enable quantification of pediatric antibiotic exposure in large aggregated datasets. Secondly, we aimed to quantify and benchmark Hungarian pediatric antibiotic use.

First we intended to examine whether a correlation existed between dosage form data and the patient’s age. Therefore, issued prescriptions were analyzed in pharmacies. As a correlation was found between the share of liquid oral antibacterial products and the rate of pediatric antibiotic prescriptions (R=0.884; p<0.001), we extrapolated this finding to a large aggregated dataset and estimated that 34.6% of prescriptions were issued for pediatric cases (95% confidence interval: 19.7–60.0). Taking into account the demography of the population, children were exposed to antibiotics three times more often than adults with a corresponding annual prescription rate of 2.6.

We demonstrated that simple drug-related data can be linked to a patient-related measure as we found strong associations between dosage form data and patients’ age. Based on this association, massive pediatric antibiotic exposure was found. Due to the general availability of dosage form data and the ease of the estimation method, the reported concept can be used to quantify pediatric antibiotic use in large aggregated datasets or when age stratification is absent.

## Introduction

Antibiotics are amongst the most commonly prescribed medicines to children [1–3]. Despite the frequent pediatric use, comprehensive data are available for only few countries [4–6]. In many countries (including Hungary), limited access to comprehensive patient-level data, due to confidentiality/privacy issues, could be a major obstacle. Apart from overcoming this limited data access, it would also be very useful to have a tool for quantifying pediatric use in large aggregated datasets (e.g. the European Surveillance of Antimicrobial Consumption, or ESAC-net database), where age stratification is missing.

As liquid dosage forms are available for many antibiotics, we hypothesized that their consumption can be linked to pediatric antibiotic use and hence drug-related data can be translated to a patient-related measure.

The goal of the present work was to report a simple estimation method to enable the quantification of pediatric antibiotic exposure. Secondly, we aimed to quantify and benchmark Hungarian pediatric antibiotic use.

## Results and Discussion

### Patient-Level Sample Data

**Fig. 1:**
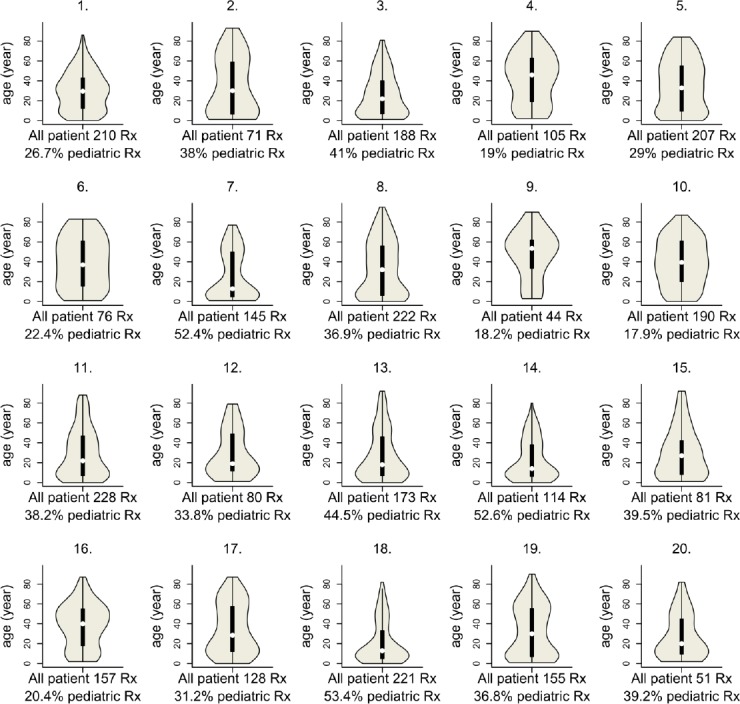
Violin plot showing the age distribution of antibiotic users in the 20 different community pharmacies

During the 120 study-days, around 50,000 prescriptions were dispensed, out of which 2,846 referred to antibacterials. In more than 90% of the cases, one package of antibacterial product was ordered per prescription, with an average of 4.3 DDDs in children and 8.1 DDDs in adults. In total, 1,009 prescriptions (36.1%) were prescribed for children (range per pharmacy: 17.9– 53.4%, see also Figure 1), with almost exclusively oral antibacterials being given out, and parenteral products being ordered only in 20 cases. Within oral antibacterials, the share of liquid oral forms ranged between 5.9% and 25.0% in different pharmacies (mean: 11.8 ± 5.1). Liquid oral antibacterials were prescribed in 651 cases, mainly for children (646 cases).

#### Linear Regression

**Fig. 2:**
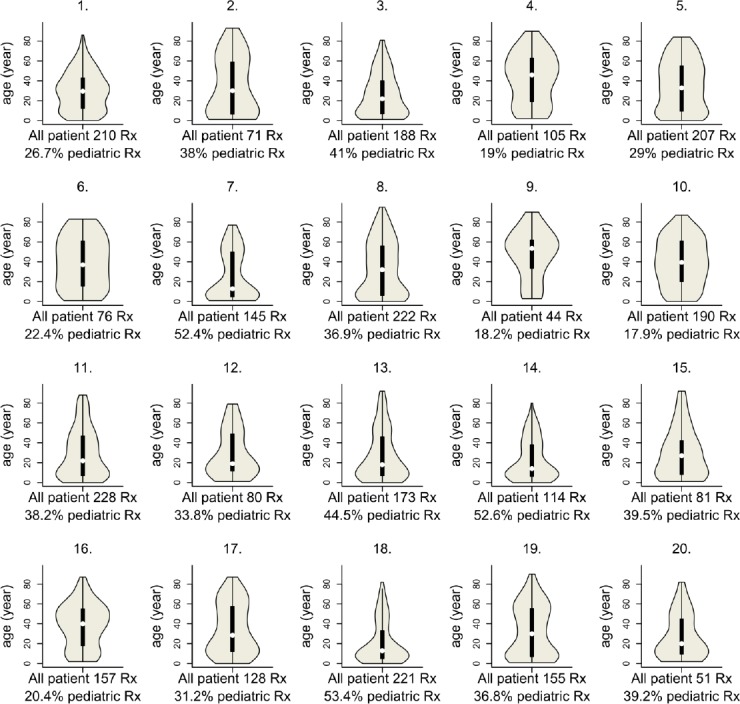
Summary of the regression model (concept and substituted values)

An association was found between the share of liquid oral antibacterial use and the rate of pediatric antibiotic prescriptions (R2=0.7806; p<0.001; see Figure 2.

### Aggregated Data Expressed in DDDs

In total, 5,126,977 DDDs of antibiotics were dispensed. Almost only oral antibacterials were prescribed (99.5%), out of which 11.5% were liquid oral formulations. Applying the result of the linear regression to the aggregated regional dosage form data, it was estimated that 34.6% (95% confidence interval: 19.7–60.0) of the antibiotic prescriptions were ordered for children, while the rest (65.4%) were prescribed for adults.

Considering the population of the children (199,434 children, 14.9% of the total population according to the Central Statistical Office) within the region and their 34.6% share from all antibiotic prescriptions, children were prescribed antibiotics three times more often than adults ((34.6/14.9)/(65.4/85.1)=3.02). Bearing in mind the weighted average DDD content (4.3 vs. 8.1 DDDs) and the proportional rate (34.6% vs. 65.4%) of pediatric and adult prescriptions, the calculated number of pediatric prescriptions were 262,165, which corresponds to an annual pediatric prescription rate of 2.6 (262,165/199,434*2). Pediatric antibiotic exposure expressed in DDD/1,000 children inhabitants/year was 31.1 (2.63*4.32*1,000/365).

In the present report, a strong association between liquid oral antibacterial use and the rate of pediatric antibiotic prescriptions is shown. The availability of comprehensive pediatric antibiotic use data are limited [7]. On the other hand, data on the use of different dosage forms are easily available in simple aggregated datasets, such as dosage form, strength, and package size, which are all included in the official trade name of the products.

Despite the relatively easy computability of the dosage form data, no publication exists on the use of different oral antibiotic formulations. Bronzwaer was the first who expressed the need for age-stratified antibiotic use data and presumed that the analysis of the liquid formulation might help in assessing pediatric use [8]. Liquid oral products are ‘age-adapted’ drug formulations. They are developed primarily for children, but also for those patients (e.g. some of the very elderly) who have difficulties safely swallowing solid oral formulations [9]. In the present study, liquid oral products were prescribed almost exclusively for children, and associations between the use of liquid oral formulations and the proportion of pediatric antibiotic prescriptions were detected.

In the literature, different measures were used to express antibiotic exposure in children [4, 10]. A prevalence rate cannot be estimated from the present work. Prescription rate was found to be 2.6 prescriptions per child inhabitant per year, while the pediatric DID (DDD/1000 children inhabitant/day) was estimated to be 31.1. Pediatric prescription rate for similar age groups (those aged under 14–17 years) ranged between 0.2 and 1.3 prescriptions per child inhabitant per year in the literature [4, 10]. The only study which calculated the pediatric DID found a value of 67 in an Italian and 35 in a Danish region for children aged 0–19 years [11].

In summary, compared to the limited pediatric antibiotic use data reported, a relatively frequent exposure of Hungarian children to antibiotics is suggested.

A major limitation of this study is the direct application of the revealed correlation coefficient to the foreign dataset. Similar associations between the use of liquid oral dosage forms and age certainly exist, but as the usage of liquid formulations and populations’ age distribution may differ, each country should set up their own linear regression model by the analysis of suitable patient-level sample data. Another handicap worth mentioning is that we calculated the annual pediatric prescription rate by duplicating the half-year data. As the study period covered the winter months, including the influenza epidemics, but excluded the start of the semester in school/kindergarden (i.e. September, October) with peaks in the incidence of respiratory tract infections, the reported annual pediatric antibiotic use value is a valid estimation. Furthermore, we have only analyzed data of one Hungarian region, not the entire country. Considering that this region covers almost 20% of the area and more than 13% of the population of Hungary, the level of antibiotic use ranks in the middle of Hungarian regions (data not shown), and it is not likely that proportional use of liquid formulations differs largely in other regions, therefore our results reflect the Hungarian situation well.

In this study, in the different sample pharmacies, the rate of pediatric prescription varied highly, and the share of liquid formulations ranged substantially. Diversity may be due to the various catchment populations of pharmacies: the population’s age distribution may differ depending on where the pharmacies are located, as for example pharmacies next to a pediatricians’ office dispense more pediatric therapies. Both diversity and the recorded similar share of liquid oral formulations (11.8% ± 5.1% in the sample pharmacies vs. 11.5% in comprehensive regional data) reflect the representativeness of the sample and validity of our estimation.

## Experimental (Methods)

First and foremost, we intended to examine the correlation between dosage form data and the patient’s age. As both of these data are available on issued prescriptions only, we conducted a study in 20 sample pharmacies. As a correlation was found between these two variables (see details below), results were projected to the regional dataset. Details of the analysis and a schematic figure (Figure 1) on the estimation method are given below.

### Patient-Level Sample Data

A manual review of prescriptions of all dispensed antibacterial products was performed retrospectively in 20 community pharmacies in the Southern Great Plain (SGP) region (total number of pharmacies = 445). The study period was between January and June 2007. Dispensed prescriptions of one workday (study-days) were reviewed for each month and for each pharmacy. The six study-days per pharmacy were selected by the double permutation method. Prescriptions pertaining to systemic antibacterials (ATC class J01) were identified. The dispensed quantity (number of sold boxes), patients’ ages, and product information (dosage form, package size) were recorded from prescriptions.

All antibacterial products were grouped into three categories: parenteral, solid oral (e.g. tablet), and liquid oral (e.g. powder for suspensions). By capturing data on patients’ ages, the rate of antibiotics prescribed for children at different ages (as a % of all antibiotic prescriptions) could be calculated. The defined daily dose (DDD) content of products was assessed (WHO ATC-DDD index, 2008) to calculate the proportional use of liquid oral antibacterials (as a % of DDDs of all oral antibacterials). The association between these variables was analyzed by linear regression (SPSS 15.0). The strongest association was found between the ratio of liquid oral dosage forms and the age under 9 years (p<0.001; R=0.936). However, as the most common definition of ‘children’ is the age under 14 years, we used this age definition and the association with a slightly weaker correlation coefficient (R=0.898) for assessing pediatric antibiotic use. For comparative purposes, the weighted average DDD (defined daily dose) content of pediatric and adult prescriptions was also determined.

### Aggregated Regional Data

For the same period, crude (i.e. number of dispensed packages) antibiotic use data for the whole region (N=445 pharmacies) was obtained from the National Health Fund Administration (NHFA). We converted data into DDDs (WHO ATC-DDD index, 2008). As in 2007, all antibiotics were reimbursed by the NHFA, all antibiotic sales in the region (covering 19.7% of the area and 13.3% of the population of Hungary) were included in the dataset. The ratio of liquid oral antibacterial use was calculated as described above. The result of the linear regression was applied to this aggregated dataset to estimate the rate of pediatric antibiotic prescriptions in the whole region. See Figure 1.

## Conclusion

We demonstrated that simple drug-related data (dosage form) can be linked to a patient-related measure (patient’s age). Based on this association, massive pediatric antibiotic exposure was found.

This methodological approach could be applied when rapid general evaluation of prescription patterns are needed in simple aggregated datasets containing data in DDD format, like the European Surveillance of Antimicrobial Consumption (ESAC-net) database. Its use would also be feasible when a population-based prescription database is not in place or not available, does not contain age-linked data, or age stratification is impossible due to privacy/confidentiality issues.
